# The non-Newtonian maxwell nanofluid flow between two parallel rotating disks under the effects of magnetic field

**DOI:** 10.1038/s41598-020-74096-8

**Published:** 2020-10-13

**Authors:** Ali Ahmadian, Muhammad Bilal, Muhammad Altaf Khan, Muhammad Imran Asjad

**Affiliations:** 1 Institute of Industry Revolution 4.0, The National University of Malaysia, 43600 UKM Bangi, Selangor Malaysia; 2 Department of Mathematics, City University of Science and Information Technology, Peshawar, Pakistan; 3 Informetrics Research Group, Ton Duc Thang University, Ho Chi Minh City, Vietnam; 4 Faculty of Mathematics and Statistics, Ton Duc Thang University, Ho Chi Minh City, Vietnam; 5 Department of Mathematics, University of Management and Technology, Lahore, Pakistan

**Keywords:** Mathematics and computing, Applied mathematics, Computer science, Scientific data

## Abstract

The main feature of the present numerical model is to explore the behavior of Maxwell nanoliquid moving within two horizontal rotating disks. The disks are stretchable and subjected to a magnetic field in axial direction. The time dependent characteristics of thermal conductivity have been considered to scrutinize the heat transfer phenomena. The thermophoresis and Brownian motion features of nanoliquid are studied with Buongiorno model. The lower and upper disk's rotation for both the cases, same direction as well as opposite direction of rotation is investigated. The subsequent arrangement of the three dimensional Navier Stoke’s equations along with energy, mass and Maxwell equations are diminished to a dimensionless system of equations through the Von Karman’s similarity framework. The comparative numerical arrangement of modeled equations is further set up by built-in numerical scheme “boundary value solver” (Bvp4c) and Runge Kutta fourth order method (RK4). The various physical constraints, such as Prandtl number, thermal conductivity, magnetic field, thermal radiation, time relaxation, Brownian motion and thermophoresis parameters and their impact are presented and discussed briefly for velocity, temperature, concentration and magnetic strength profiles. In the present analysis, some vital characteristics such as Nusselt and Sherwood numbers are considered for physical and numerical investigation. The outcomes concluded that the disk stretching action opposing the flow behavior. With the increases of magnetic field parameter $M$ the fluid velocity decreases, while improving its temperature. We show a good agreement of the present work by comparing with those published in literature.

## Introduction

The study of the fluid flow on the surface of rotating disk has got great attentions around the globe from the researcher’s due to its many applications in practical problems. Electric power generating system, rotating machinery, co rotating turbines, chemical process and computer storage, in the field of aerodynamics engineering, geothermal industry, for lubrication purposes, over the surface of rotating disk the fluid flow is widely applicable. Von Karman's^1^ examined the solution of Navier stoke's equations by considering an appropriate transformation. Further, he used the fluid flow over the rotating frame for the first time. The Von Karman's problem and its solution numerically have been discussed by Cochran^2^. Also, he used two series expansion by solving the limitation in the Von Karman's work. Sheikholeslami et al*.*^3^ used numerical technique for the solution of nanofluid flow over an inclined rotating disk. During the rotation of the disk, Millsaps and Pahlhausen^4^ studied the heat transport characteristic. The electric field in radial direction has been considered by Turkyilmazoglu^5^, where the heat transfer phenomena in magnetohydro-dynamic (MHD) fluid flow has been investigated. Under the transverse magnetic field influence, Khan et al*.*^6^ considered the non-Newtonian Powell-Eyring fluid over the rotating disk surface. The entropy generation due to porosity of rotating disk in MHD flow has been investigated by Rashidi *et al*^7^. Hayat et al*.*^8^ scrutinized the transfer of heat with viscous nanoliquid among two stretchable rotating sheets. The thermal conductivity that depends on temperature in Maxwell fluid over a rotating disk has been studied by Khan et al*.*^9^. Batchelor^10^ was the first researcher, who discussed the fluid flow between the gaps of the rotating frame. The influence of blowing with wall transpiration, suction and mixed convection has investigated by Yan and Soong^11^. Recently Shuaib *et al*^12^. studied the fractional behavior of fluid flow through a flexible rotating disk with mass and heat characteristics.

The attention of researcher’s is increasing towards nanofluid studies day by day due to its many applications in technology that binging facilities in many industrial process of heat transfer. The applications of nanofluid are in drugs delivery, power generation, micromanufactoring process, metallurgical sectors, and thermal therapy, etc. Choi^13^ is a researcher who worked for the first time on nanofluid, where he considered it for cooling and coolant purpose in technologies. He found from his work that in a base fluid (water, oil and blood, etc.) by adding the nanoparticles, the heat transfer of thermal conductivity becomes more effective. Using the idea of Choi's idea, many researchers investigated and obtained results using the nanofluids^14,15^. A concentric circular pipe with slip flow has been discussed in Turkyilmazoglu^16^. By using finite element method (FEM), Hatami et al*.*^17^ finds the solution for the heat transfer in nanofluid with free natural convective in a circular cavity. The Cattaner-Christov heat flux and thermal radiation for an unsteady squeezing MHD flow has been considered by Ganji and Dogonchi^18^. They considered the heat of transfer of the nanofluid among two plates. Dilan et al.^19^ studied nanofluids effective viscosity based on suspended nanoparticles. A carbon nanotubes based multifunctional hybrid nanoliquid has been considered by Rossella^20^. The influence of SWCNTs on human epithelial tissues is studied by Kaiser *et al*^21^. Hussanan et al*.*^22^ examined the Oxide nanoparticles for the enhancement of energy in engine nanofluids, kerosene oil and water. Saeed et al.^23^ examined nanofluid to improve the heat transfer rate and reduce time for food processing in the industry. Some recent studies related to heat and mass transfer through nanofluids are examined by many researchers^24–28^.

To study the behavior, impact and properties of magnetic field over viscous fluids is known as MHD. Salt water, plasmas and electrolytes are the examples of magnetofluids. . In the present era, the researchers and investigators are taking very keen interest in this field. A lot of work has been done in this area. The tectonic applications of MHD in engineering, chemistry, physics, industrial tackle and in many other fields, for instance, pumps, bearings, MHD generators and boundary layer control are contrived by the intercourse of conducting fluid and magnetic field. In affiliation with these applications, the work of numerous explores has been deliberated. The most essential and consequential challenge is the hydro magnetic behavior of boundary layers with the magnetic field transversely along the moving surfaces or fixed surfaces.Hannes Alfén^29^ was the first one to innovate the MHD field. In 1970 he received the Nobel Prize in physics because of his innovation in MHD field. In Medical Sciences the applications of MHD fluid flow in distinguishable configuration pertinent to human body parts are very fascinating and tectonic in the scientific area. The important applications of MHD in peristaltic flow, pulsatile flow, simple flow and drug delivery are explored by Rashidi *et al*^30^. The numerical solution has been presented by Nadeem et al.^31^ for the nanoparticles with different base fluids with slip and MHD effect. Khatsayuk et al.^32^ has explored the numerical simulation of MHD vortex technology and its verification is also ensured. The main of letters portrays casting principle into the electromagnetic mold to invoke small diameter ingots^33^. Deng and W. M. Liu et al*.*^34–37^ have presented the numerical and theoretical analysis in a rotating Bose–Einstein of the quantized vortices condensate with modulated interaction in anharmonic and harmonic potentials. They further scrutinized the nonlinear matter of the quasi-2D Bose–Einstein condensates with nonlinearity in the harmonic potential. They concluded that all of the Bose–Einstein condensates have discrete energies with an arbitrary number of localized non-linear matter waves, which are the exact solutions of the mathematical Gross-Pitaevskii equation.

Our inspiration of the present work is to analyze and model the Maxwell nano liquid flow within two stretchable coaxially rotating disks. The second priority is to initiate three dimensional Maxwell equation along with the Navier stokes equation for such type of flow and set up an arrangement for temperature, concentration, velocity and magnetic strength profile. For comparative results the built-in numerical scheme bvp4c and RK4 are opting. We have extended the idea of Ahmed ET al*.*^38^ and portrayed this mathematical model. The commitments flow factors on velocity, temperature, concentration, pressure and magnetic strength profile are studied and via graphical and in tabulated form. In the next section, the problem will be formulated and discussed.

### Mathematical formulation of the problem

**Figure 1:**
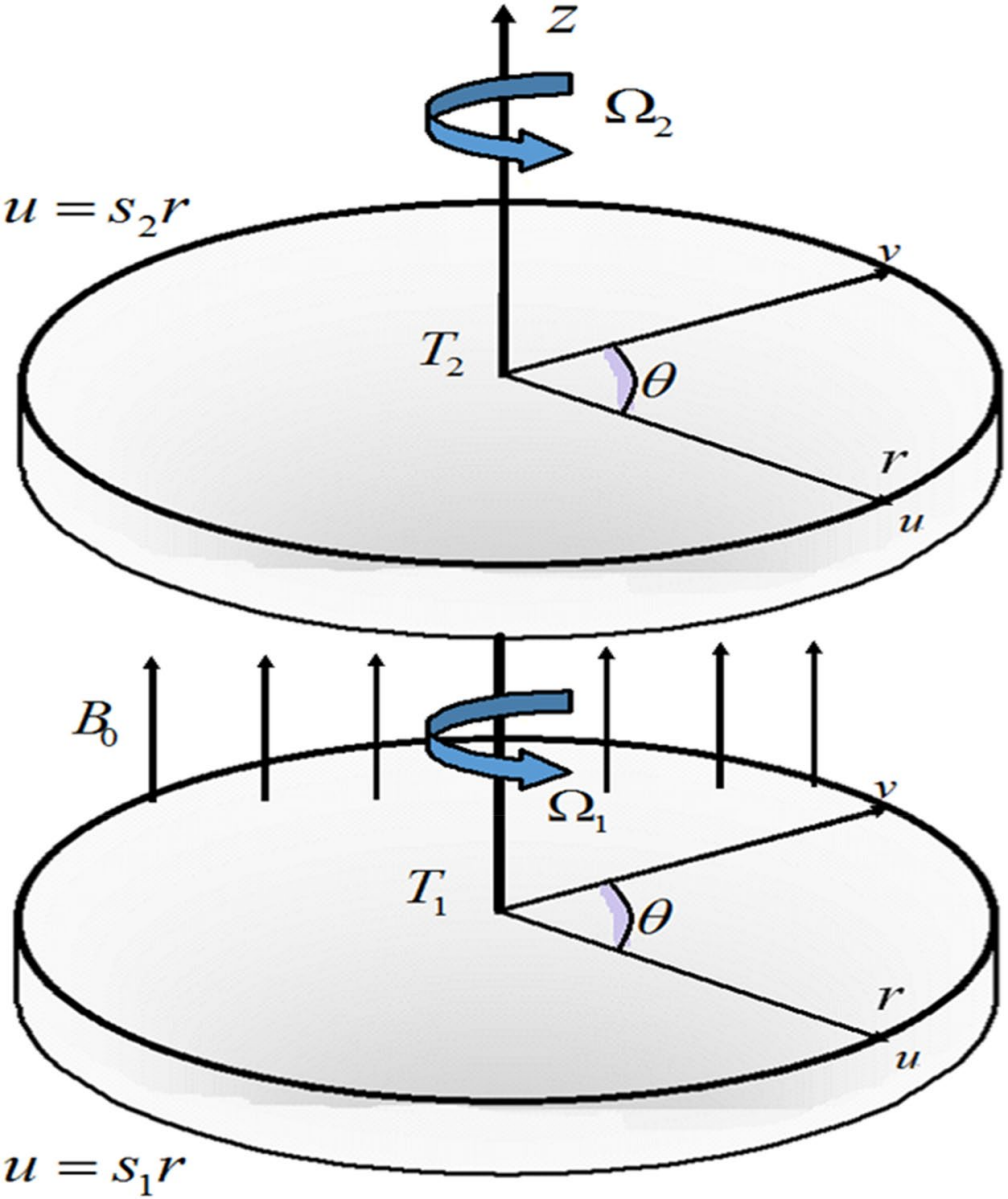
Geometry of the problem.

We assumed the nanoliquid steady motion within, the two horizontal parallel rotating disks. The disks are stretchable and subjected to magnetic field B_0_ in axial direction. The upper disk is considered at a constant position *z* = *d,* while the lower disk is at *z* = *0.* The stretching rate and velocity during rotation are $\left( {S_{1} ,\Omega_{1} } \right)$, while stretching rate and rotation velocity of upper disk are $\left( {S_{2} ,\Omega_{2} } \right)$*.* The concentration and temperature of the lower and upper disk are respectively given by $\left( {C_{1} ,C_{2} } \right)$ hand $\left( {T_{1} ,T_{2} } \right)$. The geometry of the considered problem is shown in Fig. 1*.* The governing equation of nanofluid flows are^9,39^

1 $$ \frac{\partial u}{{\partial r}} + \frac{u}{r} + \frac{\partial w}{{\partial z}} = 0, $$

2 $$ \begin{gathered} u\frac{\partial u}{{\partial r}} + w\frac{\partial u}{{\partial z}} - \frac{{u^{2} }}{r} = - \frac{1}{\rho }\frac{\partial P}{{\partial r}} + \nu (2\frac{{\partial^{2} u}}{{\partial r^{2} }} + \frac{{\partial^{2} u}}{{\partial z^{2} }} + \frac{\partial }{\partial r}(\frac{\partial w}{{\partial z}}) + \frac{2}{r}\frac{\partial u}{{\partial r}} - \frac{2u}{{r^{2} }}) \hfill \\ - \lambda_{1} (u^{2} \frac{{\partial^{2} u}}{{\partial r^{2} }} + w^{2} \frac{{\partial^{2} u}}{{\partial z^{2} }} + 2uw\frac{\partial }{\partial r}(\frac{\partial u}{{\partial z}}) - \frac{2uv}{r}\frac{\partial v}{{\partial r}} - \frac{2vw}{r}\frac{\partial v}{{\partial z}} + \frac{{uv^{2} }}{{r^{2} }} + \frac{{v^{2} }}{r}\frac{\partial u}{{\partial r}}) \hfill \\ \,\,\,\,\,\,\,\,\,\,\,\,\,\,\,\,\,\,\,\,\,\,\,\,\,\,\,\,\,\,\,\,\,\,\,\,\,\,\,\,\,\, - \frac{{\sigma^{2} }}{{B_{0} }}\rho (u + w\lambda_{1} u_{2} ), \hfill \\ \,\,\,\,\,\,\,\,\,\,\,\,\,\,\,\,\,\,\,\,\,\,\,\,\,\,\,\,\,\,\, \hfill \\ \end{gathered} $$

3 $$ \begin{gathered} u\frac{\partial v}{{\partial r}} + w\frac{\partial v}{{\partial z}} - \frac{uv}{r} = \nu (2\frac{{\partial^{2} v}}{{\partial r^{2} }} + \frac{v}{{r^{2} }} + \frac{{\partial^{2} u}}{{\partial z^{2} }} + \frac{1}{r}\frac{\partial v}{{\partial r}}) - \lambda_{1} (u^{2} \frac{{\partial^{2} v}}{{\partial r^{2} }} + w^{2} \frac{{\partial^{2} v}}{{\partial z^{2} }} \hfill \\ + 2uw\frac{\partial }{\partial r}(\frac{\partial v}{{\partial z}}) - \frac{2uv}{r}\frac{\partial v}{{\partial r}} - \frac{2vw}{r}\frac{\partial u}{{\partial z}} - 2\frac{{u^{2} v}}{{r^{2} }} + \frac{{v^{2} }}{r}\frac{\partial v}{{\partial r}}) - \frac{{\sigma^{2} }}{{B_{0} }}\rho (v + w\lambda_{1} v_{2} ), \hfill \\ \,\,\,\,\,\,\,\,\,\,\,\,\,\,\,\,\,\,\,\,\,\,\,\,\,\,\,\,\,\,\, \hfill \\ \end{gathered} $$

4 $$ \begin{gathered} u\frac{\partial w}{{\partial r}} + w\frac{\partial w}{{\partial z}} = - \frac{1}{\rho }\frac{\partial P}{{\partial z}} + \nu (\frac{\partial }{\partial r}(\frac{\partial u}{{\partial z}}) + \frac{{\partial^{2} w}}{{\partial r^{2} }} + \frac{1}{r}\frac{\partial u}{{\partial z}} + \frac{1}{r}\frac{\partial w}{{\partial r}} + 2\frac{{\partial^{2} w}}{{\partial z^{2} }}) \hfill \\ - \lambda_{1} (u^{2} \frac{{\partial^{2} w}}{{\partial r^{2} }} + \frac{{\partial^{2} v}}{{\partial z^{2} }} + 2uw\frac{\partial }{\partial r}(\frac{\partial w}{{\partial z}}) + \frac{{v^{2} }}{r}\frac{\partial w}{{\partial r}}), \hfill \\ \,\,\,\,\,\,\,\,\,\,\,\,\,\,\,\,\,\,\,\,\,\,\,\,\,\,\,\,\,\,\, \hfill \\ \end{gathered} $$

5 $$ \begin{gathered} (\rho c_{p} )_{f} (u\frac{\partial T}{{\partial r}} + w\frac{\partial T}{{\partial z}}) = \frac{k(T)}{r}\frac{\partial T}{{\partial r}} + \frac{\partial }{\partial r}(k(T)\frac{\partial T}{{\partial r}}) + \frac{\partial }{\partial r}(k(T)\frac{\partial T}{{\partial z}}) \hfill \\ + (\rho c_{p} )_{p} [D_{B} (\frac{\partial T}{{\partial z}}\frac{\partial C}{{\partial z}} + \frac{\partial T}{{\partial r}}\frac{\partial C}{{\partial r}}) + \frac{{D_{T} }}{{T_{2} }}\{ (\frac{\partial T}{{\partial z}})^{2} + (\frac{\partial T}{{\partial r}})^{2} \} ], \hfill \\ \end{gathered} $$

6 $$ u\frac{\partial C}{{\partial r}} + w\frac{\partial C}{{\partial z}} = D_{B} (\frac{{\partial^{2} C}}{{\partial r^{2} }} + \frac{1}{r}\frac{\partial C}{{\partial r}} + \frac{{\partial^{2} C}}{{\partial z^{2} }}) + \frac{{D_{B} }}{{T_{2} }}(\frac{{\partial^{2} T}}{{\partial r^{2} }} + \frac{1}{r}\frac{\partial T}{{\partial r}} + \frac{{\partial^{2} T}}{{\partial z^{2} }}), $$

7 $$ - w\frac{\partial Br}{{\partial z}} - Br\frac{\partial w}{{\partial z}} + u\frac{\partial Bz}{{\partial z}} + Bz\frac{\partial u}{{\partial z}} + \frac{1}{{\sigma \mu_{2} }}(\frac{{\partial^{2} Br}}{{\partial r^{2} }} + \frac{{\partial^{2} Br}}{{\partial z^{2} }} + \frac{1}{r}\frac{\partial Br}{{\partial r}} - \frac{Br}{{r^{2} }}) = 0, $$

8 $$ \begin{gathered} - u\frac{\partial B\theta }{{\partial r}} - B\theta \frac{\partial u}{{\partial r}} + v\frac{\partial Br}{{\partial r}} + Br\frac{\partial v}{{\partial r}} + v\frac{\partial Bz}{{\partial z}} + Bz\frac{\partial v}{{\partial z}} - w\frac{\partial B\theta }{{\partial z}} - B\theta \frac{\partial w}{{\partial z}} \hfill \\ \,\,\,\,\,\,\,\,\,\,\,\,\,\,\, + \frac{1}{\sigma \mu }_{2} (\frac{{\partial^{2} B\theta }}{{\partial r^{2} }} + \frac{{\partial^{2} B\theta }}{{\partial z^{2} }} + \frac{1}{r}\frac{\partial B\theta }{{\partial r}} - \frac{B\theta }{{r^{2} }}) = 0, \hfill \\ \end{gathered} $$

9 $$ w\frac{\partial Br}{{\partial r}} + Br\frac{\partial w}{{\partial r}} + \frac{1}{r}wBr - u\frac{\partial Bz}{{\partial r}} + Bz\frac{\partial u}{{\partial r}} - \frac{1}{r}uBz + \frac{1}{{\sigma \mu_{2} }}(\frac{{\partial^{2} Bz}}{{\partial r^{2} }} + \frac{{\partial^{2} Bz}}{{\partial z^{2} }} + \frac{1}{r}\frac{\partial Bz}{{\partial r}}) = 0, $$

where $T$ represent the fluid temperature. The nanofluid heat capacity and base fluid specific heat are $\left( {\rho C_{p} } \right)_{nf}$ and $\left( {\rho C_{p} } \right)_{f}$ respectively. The heat flux $q$ is defined as

10 $$ q = - \nabla Tk(T), $$

In which variable thermal conductivity $k\left( T \right)$ can be written as^9^

11 $$ k(T) = k_{\infty } (1 + \varepsilon \frac{{T - T_{2} }}{{T_{1} - T_{2} }}). $$

$\varepsilon$ Is the parameter of variable thermal conductivity and $k_{\infty }$ is the fluid thermal conductivity.

The boundary conditions are:

12 $$ \left. \begin{gathered} u = s_{1} r,\,\,\,\,v = \omega_{1} r,\,\,\,\,w = 0,\,\,\,\,T = T_{1} ,\,\,\,\,C = C_{1} ,\,\,\,Br = 0,\,\,\,\,Bz = 0\,\,\,\,\,\,\,\,\,\,\,\,\,\,\,\,\,\,\,\,\,at\,\,\,z = 0 \hfill \\ u = s_{2} r,\,\,\,\,v = \omega_{2} r,\,\,\,\,w = 0,\,\,\,\,T = T_{2} ,\,\,\,\,C = C_{2} ,\,Br = \frac{{dM_{0} }}{2R},Bz = - \alpha M_{0} ,\,\,\,at\,\,\,z = d. \hfill \\ \end{gathered} \right\} $$

### Transformation

The transformation, which are adopted to make the system of PDE dimensionless are as follow^38^:

13 $$ \left. \begin{gathered} u = r\Omega_{\,1} f^{\prime}(\eta ),\,\,\,v = r\Omega_{\,1} g(\eta ),\,\,\,\,w = - 2d\Omega_{\,1} f(\eta ),\,\,\,\,\eta = \frac{z}{d} \hfill \\ p = \rho \Omega_{\,1} v(P(\eta ) + \frac{1}{2}\frac{{r^{2} }}{{d^{2} }}),\,\,\,\,\Theta (\eta ) = \frac{{T - T_{1} }}{{T_{1} - T_{2} }},\,\,\,\,\phi (\eta ) = \frac{{C - C_{1} }}{{C_{1} - C_{2} }} \hfill \\ Br = r\Omega M_{0} M^{\prime}(\eta ),\,\,\,\,\,B\theta = r\Omega M_{0} N(\eta ),\,\,\,\,\,Bz = M_{0} \sqrt {(2\nu_{f} \Omega )} M(\eta ). \hfill \\ \end{gathered} \right\} $$

The required dimensionless form of the system of differential equations given in Eqs. (1–9) are:

14 $$ f^{\prime\prime\prime} = \frac{{Re((f^{\prime})^{2} - g^{2} - 2ff^{\prime\prime}) - Re(4ff^{\prime}f^{\prime\prime} - 4fgg^{\prime}) + MRe(f^{\prime} - 2\beta_{1} ff^{\prime\prime}) - \Lambda }}{{1 - 4Re\beta_{1} f^{2} }} $$

15 $$ g^{\prime\prime} = \frac{{ - 2Re(fg^{\prime} - f^{\prime}g) - Re\beta_{1} (4ff^{\prime}g^{\prime} + 4ff^{\prime\prime}g) + MRe(g - 2\beta_{1} fg^{\prime})}}{{1 - 4Re\beta_{1} f^{2} }} $$

16 $$ P{^{\prime}} = - 2f^{\prime\prime} - Re(4ff^{\prime} - 8\beta_{1} f^{2} f^{\prime\prime}), $$

17 $$ \Theta ^{\prime\prime} = \frac{{ - 2{\text{Re}} \Pr f\Theta ^{\prime} - \varepsilon \Theta ^{{\prime}{2}} - \Pr Nb\Theta ^{\prime}\Phi ^{\prime} + \Pr Nt\Theta ^{{\prime}{2}} }}{1 + \varepsilon \Theta } $$

18 $$ \Phi ^{\prime\prime} = - 2{\text{Re}} Scf\Phi ^{\prime} - \frac{Nt}{{Nb}}\Theta ^{\prime\prime}, $$

19 $$ M^{\prime\prime\prime\prime} = - 2{\text{Re}} Bt(Mf^{\prime\prime\prime} + f^{\prime\prime}M^{\prime} - fM^{\prime\prime\prime} - M^{\prime\prime}f^{\prime}), $$

20 $$ N^{\prime\prime} = 2{\text{Re}} Bt(Mg^{\prime} - fN^{\prime}), $$

with condition

21 $$ \left. \begin{gathered} f(0) = 0,\,\,\,\,f^{\prime}(0) = S_{1} ,\,\,\,\,g(0) = 1,\,\,\,\,P(0) = 1,\,\,\,\,\Theta (0) = 1,\,\,\,\,\Phi (0) = 1,\,M^{\prime}(0) = 0,\,\,\,\,N(0) = 0, \hfill \\ \,\,at\,\,\,\,\,\eta = 0 \hfill \\ f(1) = 0,\,\,\,\,f^{\prime}(1) = S_{2} ,\,\,\,\,g(1) = \Omega ,\,\,\,\,\,\,\,\,\Theta (1) = 0,\,\,\,\,\Phi (1) = 0,\,M^{\prime}(1) = 1,\,\,\,\,N(1) = 1, \hfill \\ \,\,\,\,at\,\,\,\,\,\eta = d \hfill \\ \end{gathered} \right\} $$

The magnetic field $M$, Deborah number $\beta_{1}$, lower and upper disks stretching parameters $S_{1}$ and $S_{2}$, parameter of Brownian motion $Nb$, Reynolds number ${\text{Re}}$, thermophoresis parameter $Nt$ and Schmidth number Sc are defined as:

22 $$ \begin{gathered} M = \frac{{\sigma B_{0}^{2} }}{{\rho \Omega_{1} }},\beta_{1} = \lambda_{1} \Omega_{1} ,S_{1} = \frac{{s_{1} }}{{\Omega_{1} }},S_{2} = \frac{{s_{2} }}{{\Omega_{2} }},Nb = \frac{{D_{B} \left( {C_{1} - C_{2} } \right)\left( {\rho c_{p} } \right)_{p} }}{{\nu \left( {\rho c_{p} } \right)_{f} }}, \hfill \\ Nt = \frac{{D_{B} \left( {T_{1} - T_{2} } \right)\left( {\rho c_{p} } \right)_{p} }}{{\nu T_{2} \left( {\rho c_{p} } \right)_{f} }},Re = \frac{{\Omega_{1} d^{2} }}{\nu },Sc = \frac{\nu }{{D_{B} }}. \hfill \\ \end{gathered} $$

### Sherwood and Nusselt numbers

The mass and rate of heat transfer for both disks can be illustrated as^38^:

23 $$ \begin{gathered} Sh_{r1} = - \frac{h}{{k\left( {C_{1} - C_{2} } \right)}}\left( {\frac{\partial C}{{\partial z}}} \right),Nu_{r1} = - \frac{h}{{k\left( {T_{1} - T_{2} } \right)}}\left( {\frac{\partial T}{{\partial z}}} \right),atz = 0, \hfill \\ Sh_{r2} = - \frac{h}{{k\left( {C_{1} - C_{2} } \right)}}\left( {\frac{\partial C}{{\partial z}}} \right),Nu_{r2} = - \frac{h}{{k\left( {T_{1} - T_{2} } \right)}}\left( {\frac{\partial T}{{\partial z}}} \right),atz = d. \hfill \\ \end{gathered} $$

The dimensionless form of Sherwood and Nusselt numbers can be written as

24 $$ \begin{gathered} Sh_{r1} = - \Phi^{\prime}\left( 0 \right),Nu_{r1} = - \Theta^{\prime}\left( 0 \right), \hfill \\ Sh_{r2} = - \Phi^{\prime}\left( 1 \right),Nu_{r2} = - \Theta^{\prime}\left( 1 \right). \hfill \\ \end{gathered} $$

## Graphical interpretation

### Results and discussions

**Figure 2:**
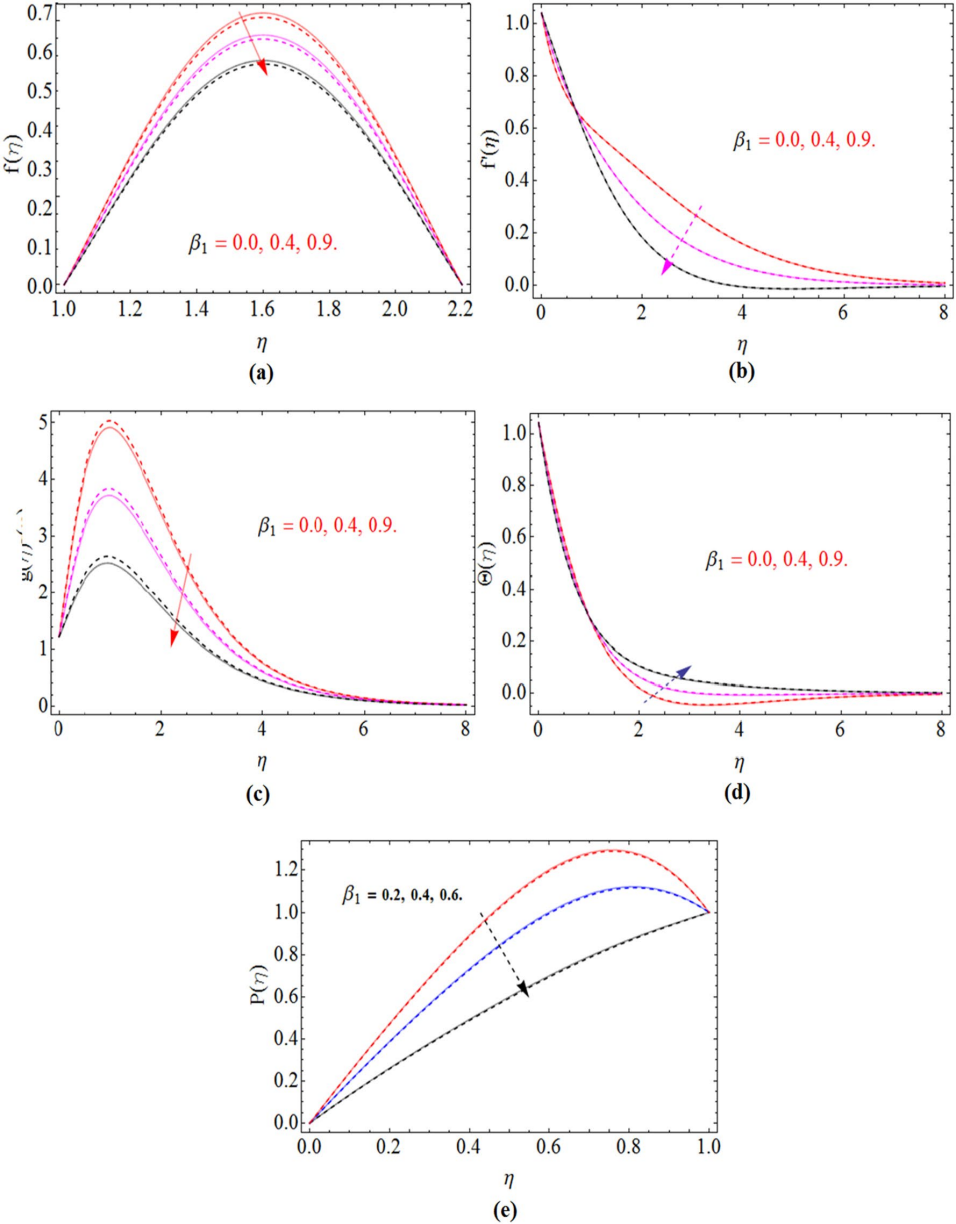
$\beta_{1}$ impact on axial $f\left( \eta \right)$, radial $f^{\prime}\left( \eta \right)$ and azimuthal velocity $g\left( \eta \right)$, temperature $\Theta \left( \eta \right)$ and pressure profile $P\left( \eta \right)$, for $S_{2}$, when $S_{1}$ = 0.0. dashed lines for $\Omega$ = 0.5 and lines for $\Omega$ =  − 0.5.

**Figure 3:**
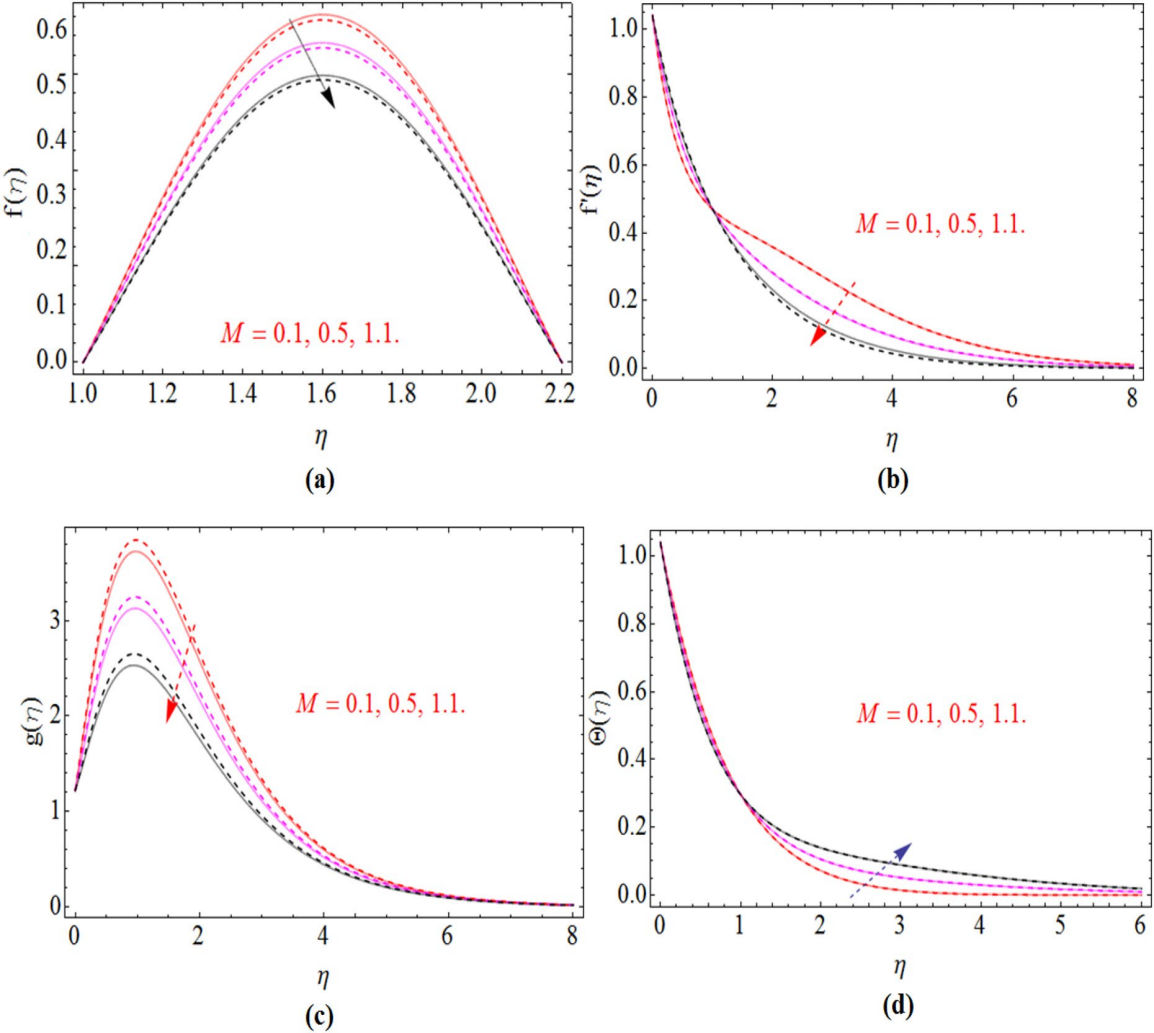
$M$ impact on an axial $f\left( \eta \right)$, radial $f^{\prime}\left( \eta \right)$ and azimuthal velocity $g\left( \eta \right)$ and temperature profile $\Theta \left( \eta \right)$, for $S_{2}$, when $S_{1}$ = 0.0. dashed lines for $\Omega$ = 0.5 and lines for $\Omega$ =  − 0.5.

**Figure 4:**
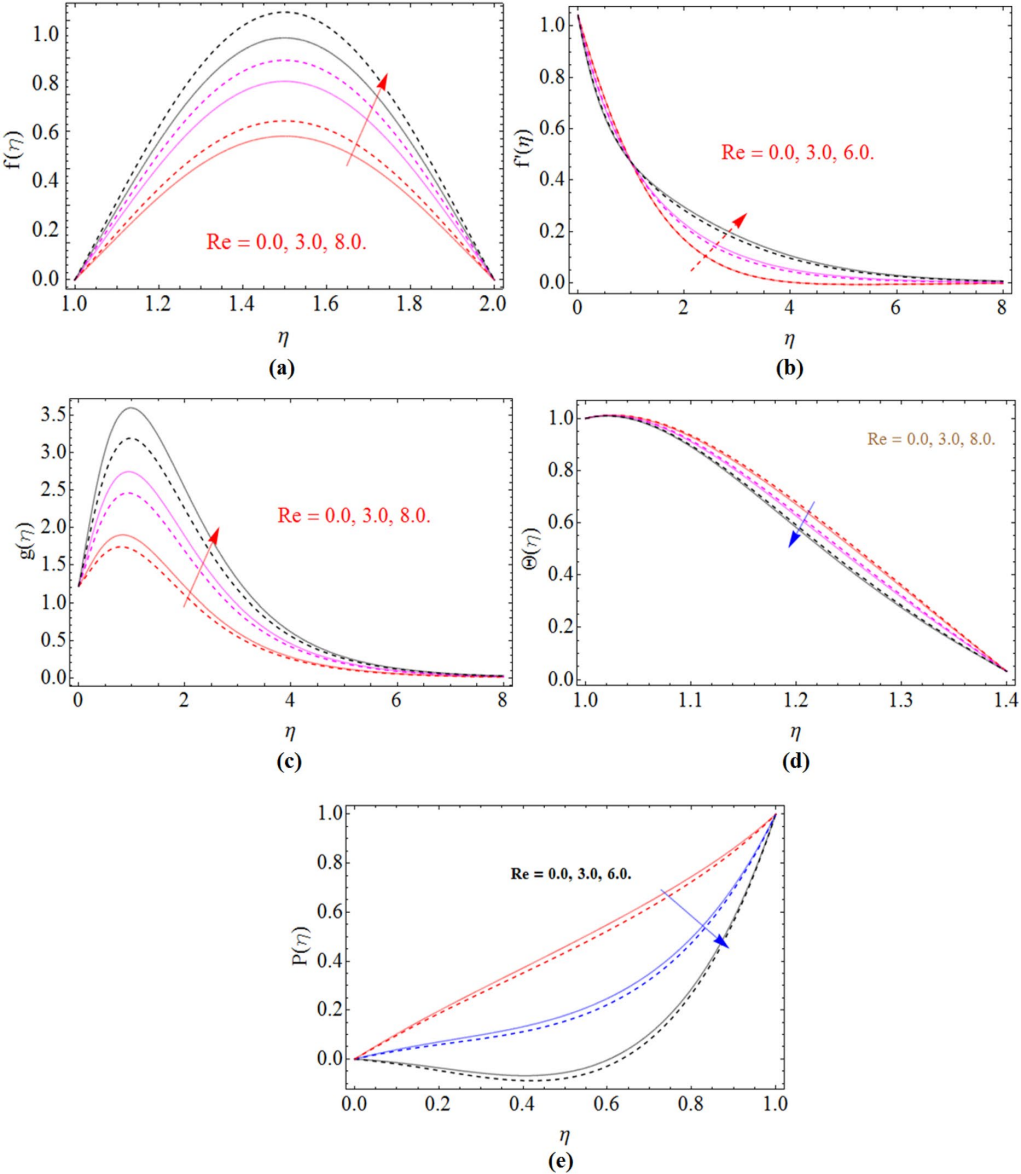
${\text{Re}}$ impact on axial $f\left( \eta \right)$, radial $f^{\prime}\left( \eta \right)$ and azimuthal velocity $g\left( \eta \right)$, temperature profile $\Theta \left( \eta \right)$ and pressure profile $P\left( \eta \right)$, for $S_{2}$, when $S_{1}$ = 0.0. dashed lines for $\Omega$ = 0.5 and lines for $\Omega$ =  − 0.5.

**Figure 5:**
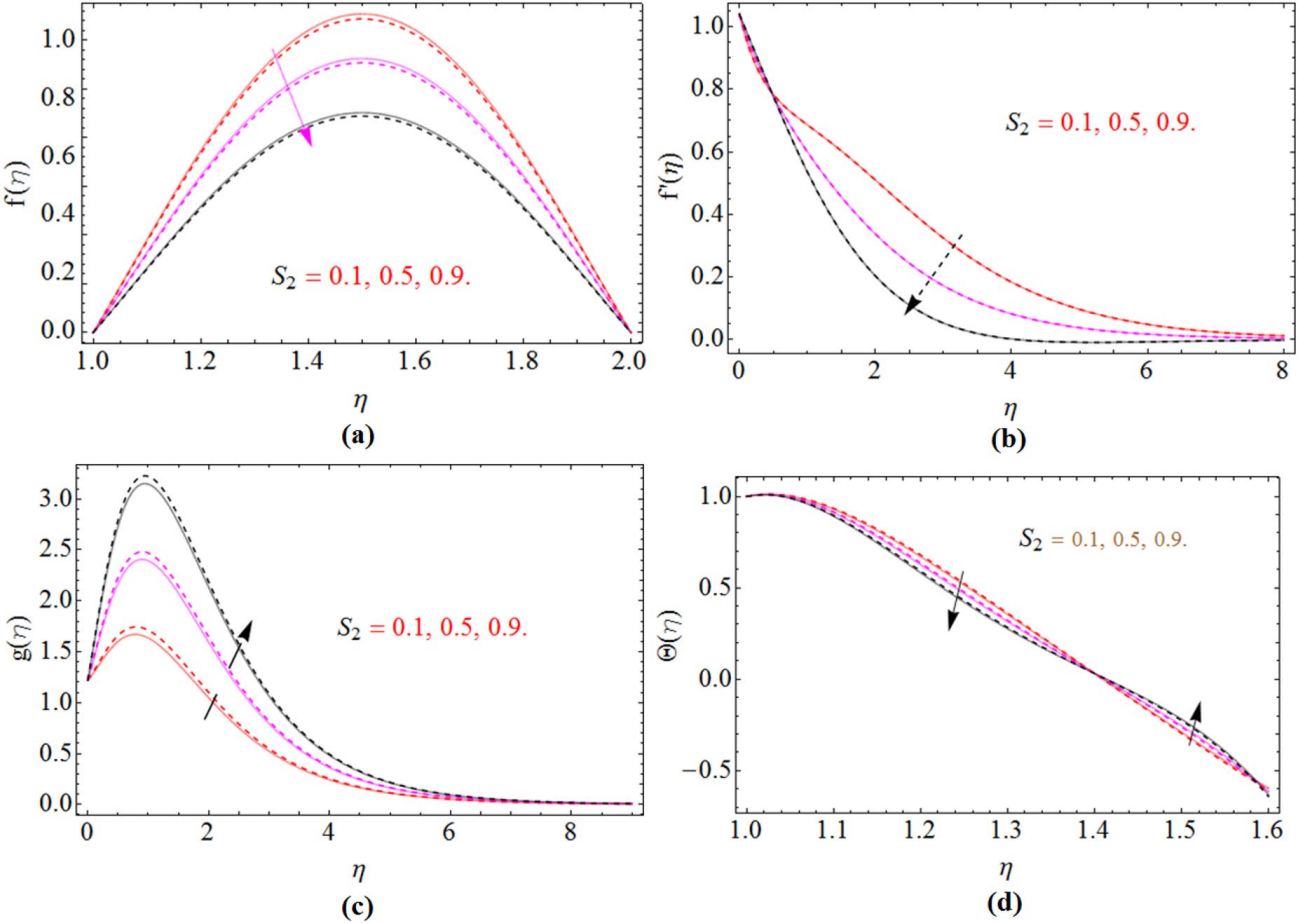
$S_{2}$ impact on axial $f\left( \eta \right)$, radial $f^{\prime}\left( \eta \right)$ and azimuthal velocity $g\left( \eta \right)$ and temperature profile $\Theta \left( \eta \right)$, for $S_{2}$, when $S_{1}$ = 0.0. dashed lines for $\Omega$ = 0.5 and lines for $\Omega$ =  − 0.5.

**Figure 6:**
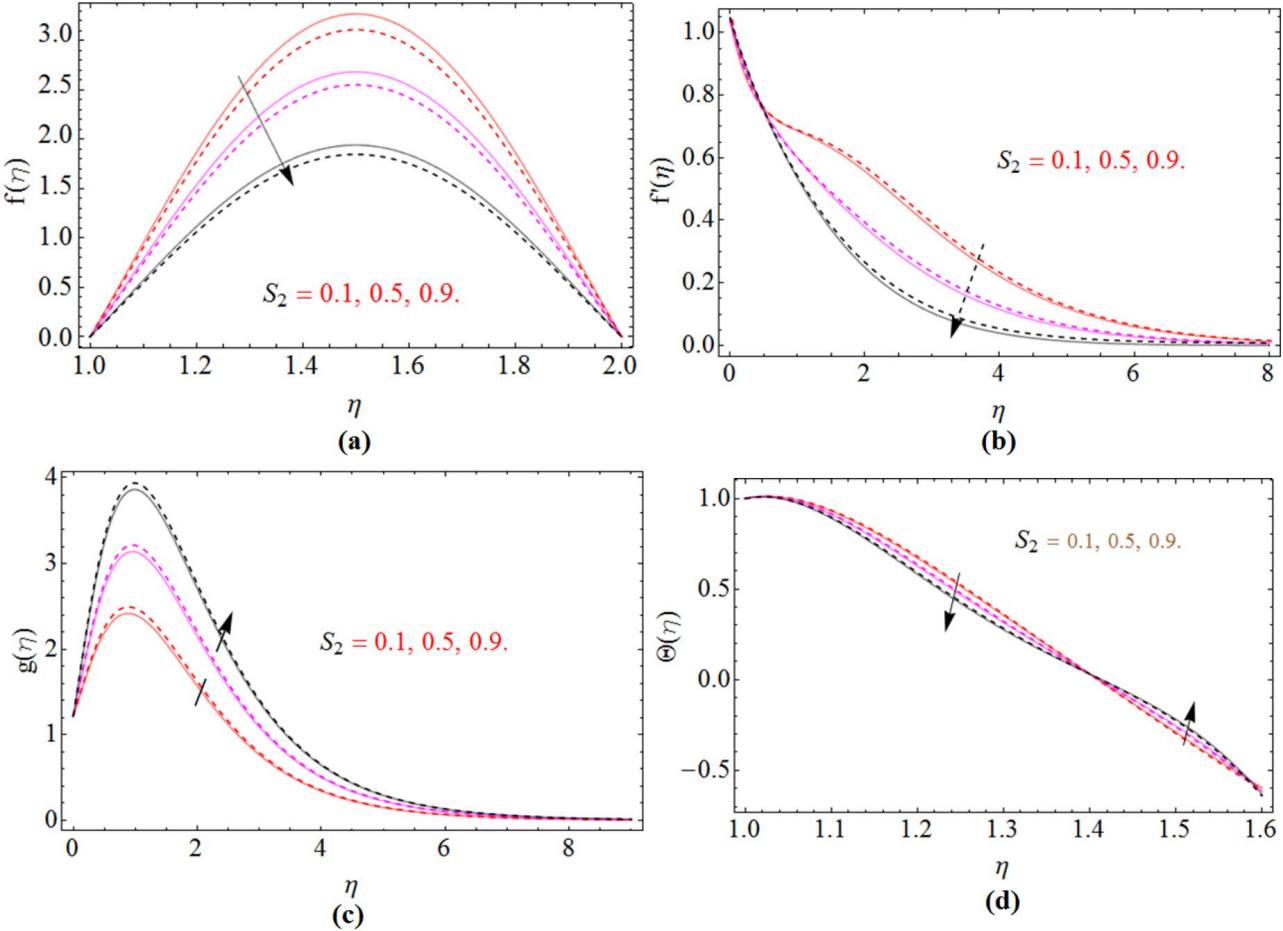
$S_{2}$ impact on axial $f\left( \eta \right)$, radial $f^{\prime}\left( \eta \right)$ and azimuthal velocity $g\left( \eta \right)$ and temperature profile $\Theta \left( \eta \right)$, for $S_{2}$, when $S_{1}$ = 0.5. dashed lines for $\Omega$ = 0.5 and lines for $\Omega$ =  − 0.5.

**Figure 7:**
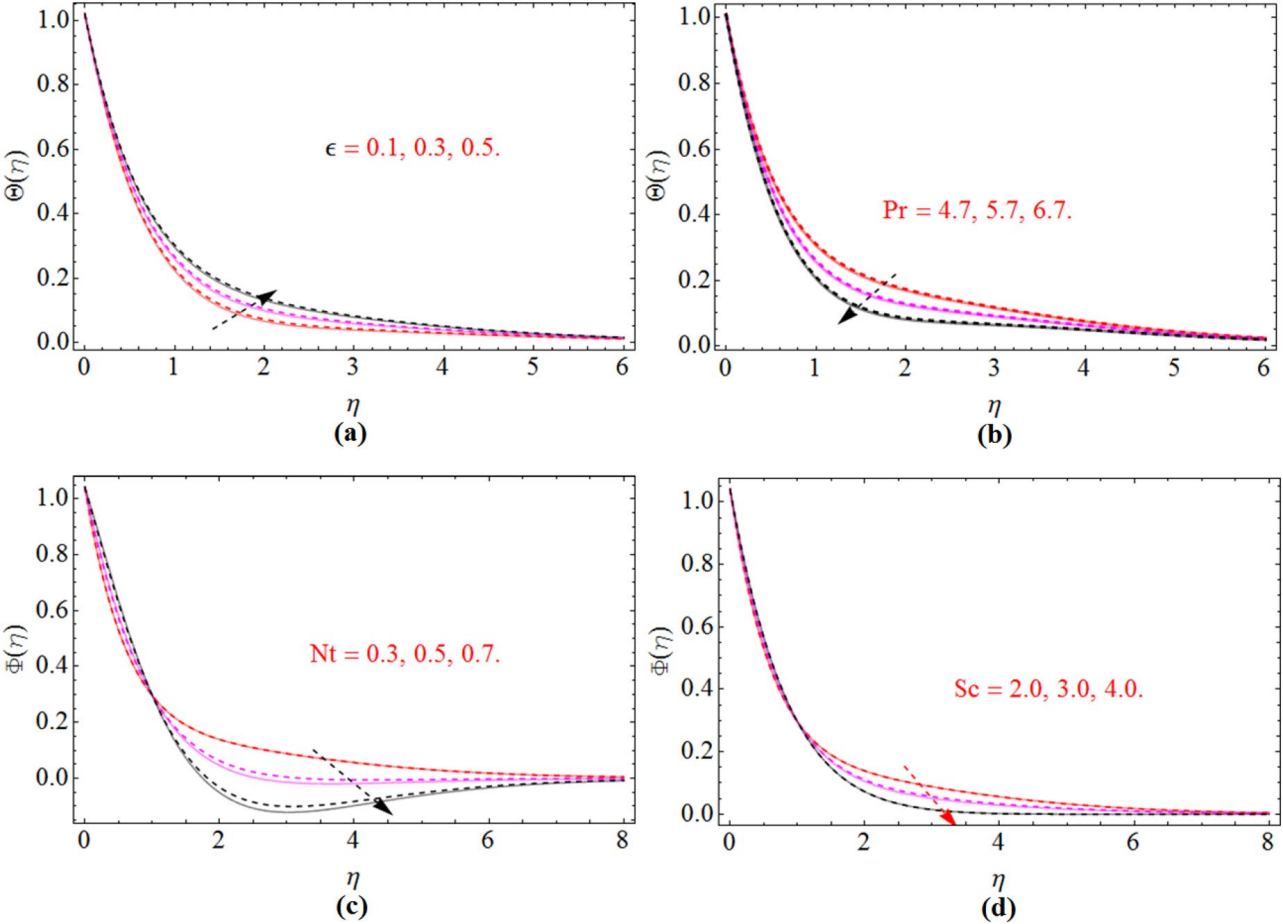
$\varepsilon$ and *Pr* impact on temperature profile $\Theta \left( \eta \right)$, while *Nt* and *Sc* on concentration profile, for $S_{2}$, when $S_{1}$ = 0.0. dashed lines for $\Omega$ = 0.5 and lines for $\Omega$ =  − 0.5.

**Figure 8:**
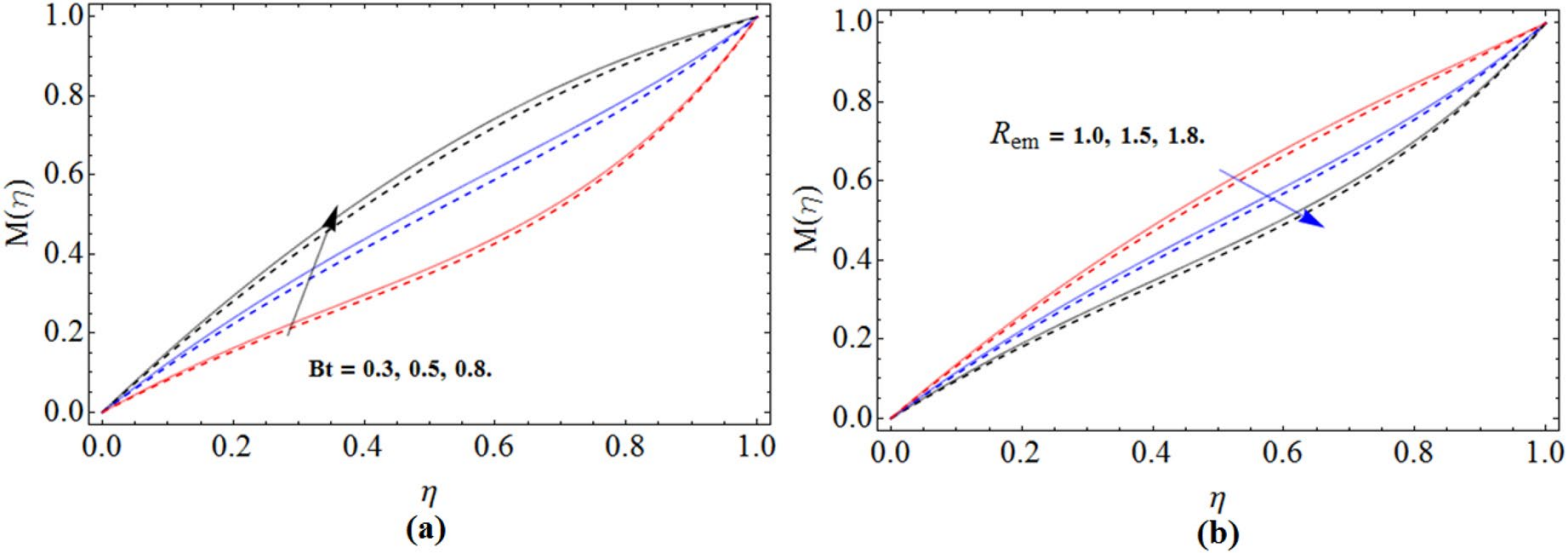
$Bt$ and $R_{em}$ impact on magnetic strength profile $M\left( \eta \right)$ for $S_{2}$, when $S_{1}$ = 0.0. dashed lines for $\Omega$ = 0.5 and lines for $\Omega$ =  − 0.5.

The governing equations of Non-Newtonian Maxwell nanofluid flow problem has been solved numerically using bvp4c scheme after using Karman’s scaling approach. In this section the results are illustrated through tables and Figures to visualize the impact of different physical constraints on velocity, pressure, concentration, temperature and magnetic strength profile. Both cases of disks rotation, same $\left( {\Omega = 0.5} \right)$ and in opposite direction $\left( {\Omega = - 0.5} \right)$ of rotation has been sketched in Figs. 2, 3, 4, 5, 6, 7, 8. The entire calculation has been performed by keeping the values of constraints as ${\text{Re}}$ = 4.0, $M$ = 0.3, $Nb = Nt$ = 0.3, $\beta_{1}$ = 0.2, $S_{1} = S_{2}$ = 0.4, $\varepsilon$ = 0.1 and $Sc$ = 3.0.

Figure 2a–e are plotted, in order to illustrate the influence of Deborah number $\beta_{1}$ on axial velocity profile $f\left( \eta \right)$, radial $f^{\prime}\left( \eta \right)$ and azimuthal velocity $g\left( \eta \right)$, temperature $\Theta \left( \eta \right)$ and pressure profile $P\left( \eta \right)$ respectively. The fluid behaves as a solid substance with high Deborah number $\beta_{1}$ shown in Fig. 2a. That’s why axial velocity reduces with the increases of $\beta_{1}$. The fluid with low Deborah number possess less elastic property and vice versa illustrated in Fig. 2b,c. So the radial velocity and azimuthal velocity reduces with the improvement of $\beta_{1}$. The fluid temperature is rises with $\beta_{1}$ shown in Fig. 2d. The pressure profile of fluid decline with the rising values of Deborah number $\beta_{1}$ Fig. 2e.

Figure 3a–d demonstrate the behavior of axial velocity profile $f\left( \eta \right)$, radial $f^{\prime}\left( \eta \right)$, azimuthal velocity $g\left( \eta \right)$ and the temperature $\Theta \left( \eta \right)$ versus magnetic parameter *M*. The axial velocity and radial velocity decline with the effects of magnetic parameter $M$ see Fig. 3a,b. Because the magnetic field creates some resistive forces, which oppose the fluid velocity and as a result axial and radial velocity reduces. The same trend has been received of azimuthal velocity via *M* Fig. 3c. By the enhancement of magnetic strength on the fluid flow generate friction, which produces some amount of heat and as a result the average temperature of the fluid increases which is given in Fig. 3d.

The dominance of Reynolds number against axial velocity, radial and azimuthal velocity is elaborated in Fig. 4a–c. Figure 4d elaborated to observe that the temperature field decline with the rising credit of Reynolds number $\left( {\text{Re}} \right)$. The pressure profile of fluid also decline with the rising values of Reynolds number Fig. 4e.

The two different cases for $S_{2}$, when the lower disk stretching rate is $\left( {S_{1} = 0} \right)$ and when it is $\left( {S_{1} = 0.5} \right)$ have been sketched in Figs. 5a,b and 6a,b. In both cases the axial and radial velocity of fluid decreases with the improving values of $S_{2}$. While in azimuthal velocity an opposite seen has been observed, because by increasing stretching rate $S_{2}$ the kinematics energy of fluid increases which enhanced the azimuthal velocity $g\left( \eta \right)$ illustrated in Figs. 5c and 6c. Figures 5d and 6d are sketched to observe the upper disk stretching impact versus temperature profile, while keeping the lower disk stretching rate $\left( {S_{1} = 0} \right)$ and $\left( {S_{1} = 0.5} \right)$ respectively. When the disk stretch the fluid particle above the disk surface get some space and become relaxed for a while, as a result their temperature reduce, which causes the average temperature of fluid to reduce.

Figure 7a,b is drawn in order to reveal the impact of the parameters $\varepsilon$ and *Pr* which represent respectively thermal conductivity and Brandt number on temperature field $\Theta \left( \eta \right)$. From Fig. 7a, it is obvious that by increasing the thermal conductivity parameter $\varepsilon$, the temperature field will improve. Figure 7b demonstrate the inverse relation of Prandtl number Pr versus temperature profile, physically large Prandtl fluid have less thermal diffusivity while less Prandtl fluid have always high thermal diffusivity, that’s why the temperature field and Prandtl number has inverse relation. Figure 7c,d are plotted to examine the influence of thermophoresis parameter $Nt$ and Schmidth number $Sc$ on $\Phi \left( \eta \right)$. The mass transfer rate reduces with the improvement of both thermophoresis parameter $Nt$ and Schmidth number $Sc$.

The dominant behavior of Batchlor number $Bt$ versus magnetic field has been illustrated in Fig. 8a. When Batchlor number is large, a less current will generates high induced magnetic field $M\left( \eta \right)$, while the opposite trend has been observed with the magnetic Reynolds number on magnetic field in Fig. 8b. The enhancement of Reynolds number reduces the magnetic fields $M\left( \eta \right)$.

Table 1 shows the comparison of our work with that in Turkyilmazoglu^12^, Ahmed et al.^38^ and Rogers and Lance^40^ for different values of rotation parameter $\Omega$, in case when $S_{1} = S_{2} = 0$. Table 2 is displayed for numerical outcomes of Reynolds number and rotation parameter $\Omega$, while keeping the upper plate stretching rate $S_{2} = 0$ and lower plate $S_{1} = 0.5$. The results in Table 2 are also compared with published work^12^. For the validity of the results two well-known best numerical approaching techniques Runge Kutta order four method and boundary value solver are compared in Table 3. The numerical outputs for Sherwood number $Sh_{r1}$ and Nusselt number $Nu_{r1}$ at lower disk are plotted in Table 3. By varying Prandtl number, thermal conductivity, magnetic field, Reynolds number, thermophoresis and upper disk stretching parameters, the Nusselt number for lower $Nu_{r1}$ and upper disks $Nu_{r2}$ are also calculated. In Table 4. the Nusselt number for lower $Nu_{r1}$ and upper disks $Nu_{r2}$ are calculated by varying Prandtl number, thermal conductivity, magnetic field, Reynolds number, thermophoresis and upper disk stretching parameters.

**Table 1:** For various valued of rotation parameter $\Omega$ the comparison of $- G^{\prime}\left( 0 \right), - F^{\prime\prime}\left( 0 \right)$ and $\Lambda$ has been shown for the case when $S_{2} = S_{1} = 0.$

$\Omega$	− _1.0_	− 0.8	− 0.3	0.0	0.50
$$F^{\prime\prime}\left( 0 \right)$$					
Ref.^40^	0.06667000	0.08384000	0.10385000	0.09987000	0.06653000
Ref.^12^	0.06667313	0.08384206	0.10385088	0.09987221	0.06653419
Ref.^38^	0.06667358	0.08384164	0.10385000	0.09987146	0.06653400
Present	0.06667723	0.08384354	0.10386000	0.09988248	0.06653500
$$- G^{\prime}\left( 0 \right)$$					
Ref.^40^	2.00094000	1.80258000	1.30432000	1.00438000	0.50251000
Ref.^12^	2.00094215	1.80257847	1.30432355	1.00437756	0.50251351
Ref.^38^	2.00094200	1.80257800	1.30432300	1.00437700	0.50251350
Present	2.00095250	1.80259000	1.30433300	1.00438600	0.50251750
$$\Lambda$$					
Ref.^40^	0.19993000	0.17184000	0.20636000	0.29924000	0.57458000
Ref.^12^	0.19992538	0.17185642	0.20635721	0.29923645	0.57457342
Ref.^38^	0.19992651	0.17185728	0.20635898	0.29923784	0.57457377
Present	0.19997752	0.17186723	0.20635981	0.29923843	0.57457499

**Table 2:** The comparison of $- G^{\prime}\left( 0 \right), - F^{\prime\prime}\left( 0 \right)$ and $\Lambda$ for different values of ${\text{Re}}$ and $\Omega$ in case, when $S_{1} = 0.5,S_{2} = 0.0.$

${\text{Re}}$	$\Omega$	$F^{\prime\prime}\left( 0 \right)$		$- G^{\prime}\left( 0 \right)$	
		Ref.^12^	Present	Ref.^12^	Present
0	− 0.5	− 2.00000007	− 2.00000000	1.50000000	1.50000000
10	− 0.5	− 1.60562889	− 1.60563754	3.40116128	3.40117328
0	0.0	− 2.00000007	− 2.00000000	1.00000000	1.00000000
10	0.0	− 1.44561724	− 1.44561896	2.56217438	2.56218932
0	0.5	− 2.00000007	− 2.0000000	0.50000000	0.50000000
10	0.5	− 1.89459839	− 1.89459945	1.50020105	1.50022800

**Table 3:** The comparison of $RK4$ and Bvp4c for Sherwood $Sh_{r1}$ and Nusselt number $Nu_{r1}$ at the lower disk, when $M = 1.2,Sc = 3.0,\Pr = 3.0,Nt = Nb = 0.3,{\text{Re}} = 4.0,\beta_{1} = 0.2,S_{1} = S_{2} = 0.5.$

$Sh_{r1}$			$Nu_{r1}$		
$\Pr$	Bvp4c	$RK4$	$Sc$	Bvp4c	$RK4$
2.0	0.8718294	0.8718295	2.0	1.557996	1.557995
3.0	0.7999895	0.7999895	2.5	1.631221	1.631220
4.0	0.7290099	0.7290098	3.0	1.696949	1.696949
5.0	0.6632382	0.6632380	3.5	1.785639	1.785639

**Table 4:** The Nusselt numbers at lower $Nu_{r1}$ and upper $Nu_{r2}$ disks respectively, when $S_{1} = 0.5,\Omega = 0.5,\beta_{1} = 0.2,Sc = 1.0,Nb = 0.3.$

$S_{2}$	$M$	${\text{Re}}$	$\Pr$	$\varepsilon$	$Nt$	$Nu_{r1}$	$Nu_{r2}$
0.0	2.0	6.0	2.5	0.5	**0.2**	0.969396	1.444753
0.3						0.829980	1.906798
0.6						0.715537	2.419063
0.0	1.0					0.996962	1.393135
	1.5					0.982571	1.422293
	2.0					0.969396	1.444753
	2.0	0.0				0.722765	1.828593
		3.0				0.867816	1.586953
		6.0				0.922574	1.379949
		6.0	1.0			0.957529	1.3392912
			3.0			0.982961	1.486556
			5.0			0.997661	1.569729
			2.0	0.0		1.149913	1.069889
				0.3		0.926457	1.335418
				0.6		0.947561	1.498999
				0.5	0.2	0.969396	1.444753
					0.4	0.848575	1.682722
					0.6	0.739949	0.938282

## Conclusion

The present numerical model is intended to explore the behavior of Non-Newtonian (Maxwell) nanoliquid moving within two stretchable rotating disks subjected to axial magnetic field. The disks are separated from each other by fixed distance. The time dependent characteristics of thermal conductivity have been considered to scrutinize the heat transfer phenomena. The thermophoresis and Brownian motion features of nanoliquid are studied with Buongiorno model. The system of equations is solved numerically through Runge Kutta order four method and bvp4c. The concluded outputs are listed as:

- The rising credit of thermophoresis and Brownian motion positively affects the temperature field.
- It is examined that by varying the upper disk stretching, the axial flow changes its behavior to upper form lower disk.
- A significant change in tangential velocity and slight enhancement in temperature profile are observed with the rising values of upper disk stretching rate.
- The temperature field is enhanced with the variation in thermal conductivity and magnetic field parameters.
- The transfer of mass and heat rate is inclined at the lower disk surface with the Schmidth number.
- When the upper disk stretching rate become zero, the heat transport rate decline at lower disk surface, while incline at upper disk with the parameter $\varepsilon$ (thermal conductivity).
- The radial, axial and azimuthal velocity decreases while temperature field increases with varying of $\beta_{1}$ (Deborah number).

## Appendix

### Solution methodology

For the solution of the model numerically, we convert the high order system into a system of first order system of differential equations which can be easily solved by the method of Runge–Kutta order four schemes. In order to convert the system the following scales are considered:$$\begin{gathered} \chi_{1} = f,\chi_{2} = f^{\prime},\chi_{3} = f^{\prime\prime},\chi_{4} = g,\chi_{5} = g^{\prime},\chi_{6} = P,\chi_{7} = \theta ,\chi_{8} = \theta ^{\prime},\chi_{9} = \phi ,\chi_{10} = \phi ^{\prime}, \hfill \\ M = \chi_{11} ,\,\,M^{\prime} = \chi_{12} ,M^{\prime\prime} = \chi_{13} ,M^{\prime\prime\prime} = \chi_{14} ,N = \chi_{15} ,N^{\prime} = \chi_{16} . \hfill \\ \end{gathered}$$

25 $$ \begin{gathered} \chi_{1}{^{\prime}} = \chi_{2} ,\chi_{2}{^{\prime}} = \chi_{3} , \hfill \\ \chi_{3}{^{\prime}} = \frac{{Re(\chi_{2}^{2} - \chi_{4}^{2} - 2\chi_{1} \chi_{3} ) - Re(4\chi_{1} \chi_{2} \chi_{3} - 4\chi_{1} \chi_{4} \chi_{5} ) + M_{1} Re(\chi_{2} - 2\beta_{1} \chi_{1} \chi_{3} ) - \Lambda }}{{1 - 4Re\beta_{1} \chi_{1}^{2} }}, \hfill \\ \chi_{4}{^{\prime}} = \chi_{5} , \hfill \\ \chi_{5}{^{\prime}} = \frac{{ - 2Re(\chi_{1} \chi_{5} - \chi_{2} \chi_{4} ) - Re\beta_{1} (4\chi_{1} \chi_{2} \chi_{5} - 4\chi_{1} \chi_{4} \chi_{3} ) + M_{1} Re(\chi_{4} - 2\beta_{1} \chi_{1} \chi_{5} ) - \Lambda }}{{1 - 4Re\beta_{1} \chi_{1}^{2} }}, \hfill \\ \chi_{6}{^{\prime}} = - 2\chi_{3} - Re(4\chi_{1} \chi_{2} - 8\beta_{1} \chi_{1}^{2} \chi_{3} ),\chi_{7}{^{\prime}} = \chi_{8} \hfill \\ \chi_{8}{^{\prime}} = \frac{{ - 2RePr\chi_{1} \chi_{8} - \varepsilon \chi_{8}^{2} - PrNb\chi_{8} \chi_{10} + PrNt\chi_{8}^{2} }}{{1 + \varepsilon \chi_{7} }}, \hfill \\ \chi_{9}{^{\prime}} = \chi_{10} , \hfill \\ \chi_{10}{^{\prime}} = - 2ReSc\chi_{1} \chi_{10} - \frac{Nt}{{Nb}}\chi_{8}{^{\prime}} , \hfill \\ \chi_{11}{^{\prime}} = \chi_{12} ,\chi_{12}{^{\prime}} = \chi_{13} ,\chi_{13}{^{\prime}} = \chi_{14} , \hfill \\ \chi_{14}{^{\prime}} = - 2ReBt(\chi_{11} \chi_{3}{^{\prime}} + \chi_{3} \chi_{12} - \chi_{1} \chi_{14} - \chi_{13} \chi_{2} ), \hfill \\ \chi_{15}{^{\prime}} = \chi_{16} ,\chi_{16}{^{\prime}} = - 2ReBt(\chi_{11} \chi_{5} - \chi_{1} \chi_{16} ). \hfill \\ \end{gathered} $$

The transform conditions are:

26 $$ \left. \begin{gathered} \chi_{1} (0) = 0,\,\,\,\,\chi_{2} (0) = S_{1} ,\,\,\,\,\chi_{4} (0) = 1,\,\,\,\,\chi_{6} (0) = 1,\,\,\,\,\chi_{7} (0) = 1,\,\,\,\,\chi_{9} (0) = 1,\,\chi_{12} (0) = 0,\,\,\,\,\chi_{15} (0) = 0,\, \hfill \\ \,at\,\,\,\,\,\eta = 0 \hfill \\ \chi_{1} (1) = 0,\,\,\,\,\chi_{2} (1) = S_{2} ,\,\,\,\,\chi_{4} (1) = \Omega ,\,\,\,\,\,\chi_{7} (1) = 0,\chi_{9} (1) = 0,\,\chi_{12} (1) = 1,\,\,\,\,\chi_{15} (1) = 1, \hfill \\ \,at\,\,\,\,\,\eta = d \hfill \\ \end{gathered} \right\} $$

## List of symbols

*ε*: Thermal conductivity
Λ: Pressure gradient parameter
*g*: Transform azimuthal velocity
*q*: Heat flux
σ: Fluid electric conductivity
*η*: Dimensionless variable
*Rd*: Radiation parameter
Ω_1_: Rotation rate of lower disk
*D*_*B*_: Brownian diffusion coefficient
*K*(*T*): Variable thermal conductivity
*ρ*: Fluid density
*λ*_1_: Relaxation time parameter
*Nb*: Brownian motion parameter
*D*_*B*_: Brownian diffusion coefficient
*V*: Kinematic viscosity
*T*: Temperature
*D*_*T*_: Coefficient of thermophoretic diffusion
*Sc*: Schmidt number
*T*_1_: Temperature of Lower disk
*Bt*: Batclor number
Φ,Θ: Transform concentration & temperature
*r*, Θ, *z*: Cylindrical coordinate system
*C*_*p*_: Specific heat
*M*: Magnetic parameter
*P*: Pressure
Ω: Rotation parameter
*d*: Vertical distance between disks
*J*: Current density
*B*_0_: Strength of magnetic field
Ω_2_: Rotation rate of upper disk
Nabla: Nabla
*Pr*: Prandtl number
*C*: Concentration
*β*_1_: Deborah number
*Nt*: Thermophoresis parameter
*D*_*T*_: Thermophoretic diffusion coefficient.
*μ*: Dynamic viscosity
***V***: Velocity vector
*k*: Thermal conductivity
Re: Reynolds number
*T*_2_: Temperature of upper disk
*R*_*em*_: Magnetic Reynolds number
*f*, *f*′: Transform axial and radial velocity
u, v, w: Velocity components
